# Comparative genomic analysis of Vibrios yields insights into genes associated with virulence towards *C. gigas* larvae

**DOI:** 10.1186/s12864-020-06980-6

**Published:** 2020-08-31

**Authors:** Hanna Kehlet-Delgado, Claudia C. Häse, Ryan S. Mueller

**Affiliations:** 1 Department of Microbiology, Oregon State University, Corvallis, Oregon, 97331 USA; 2 Carlson College of Veterinary Medicine, Oregon State University, Corvallis, Oregon, USA

**Keywords:** *Vibrio*, Aquaculture, Comparative genomics, Oyster larvae, *Vibrio coralliilyticus*, Vibriosis, *Crassostrea gigas*, Prokaryotic genomics

## Abstract

**Background:**

Vibriosis has been implicated in major losses of larvae at shellfish hatcheries. However, the species of *Vibrio* responsible for disease in aquaculture settings and their associated virulence genes are often variable or undefined. Knowledge of the specific nature of these factors is essential to developing a better understanding of the environmental and biological conditions that lead to larvae mortality events in hatcheries. We tested the virulence of 51 *Vibrio* strains towards Pacific Oyster (*Crassostreae gigas*) larvae and sequenced draft genomes of 42 hatchery-associated vibrios to determine groups of orthologous genes associated with virulence and to determine the phylogenetic relationships among pathogens and non-pathogens of *C. gigas* larvae.

**Results:**

*V. coralliilyticus* strains were the most prevalent pathogenic isolates. A phylogenetic logistic regression model identified over 500 protein-coding genes correlated with pathogenicity. Many of these genes had straightforward links to disease mechanisms, including predicted hemolysins, proteases, and multiple Type 3 Secretion System genes, while others appear to have possible indirect roles in pathogenesis and may be more important for general survival in the host environment. Multiple metabolism and nutrient acquisition genes were also identified to correlate with pathogenicity, highlighting specific features that may enable pathogen survival within *C. gigas* larvae.

**Conclusions:**

These findings have important implications on the range of pathogenic *Vibrio* spp. found in oyster-rearing environments and the genetic determinants of virulence in these populations.

## Background

The *Vibrio* genus represents a group of microorganisms ubiquitous in temperate marine, freshwater, and estuarine environments [1–3]. Members of this genus are known to have roles as commensal or pathogenic members of marine animal microbiomes [4, 5], or as free-living members of the environment [6, 7]. Incidences of diseases attributed to *Vibrio* bacteria are broadly referred to as “vibriosis”, with the identities of the etiological agents and their pathogenic mechanisms varying between hosts. In aquaculture settings, vibriosis outbreaks can lead to large economic and productivity impacts [8]. Commercial shellfish hatcheries are impacted by disease outbreaks attributed to vibrios, which can result in large mass mortalities of larvae [9–12].

Genomic sequencing of marine pathogens has aided our understanding of the genetic mechanisms of virulence in some of these systems. Within some species of *Vibrio*, there are well-defined mechanisms for virulence. For instance, within the Splendidus clade of *Vibrios*, numerous virulence factors, including an exported protein of unknown function, gene *r*5.7 [13, 14], species-specific Type 6 Secretion System effectors [15], and the Multifunctional-Autoprocessing Repeats-in-Toxin (MARTX) cluster [14], have been shown to affect Pacific Oyster (*Crassostrea gigas*) adults and juveniles. The importance of a zinc metalloprotease has also been reported in several *Vibrio* oyster pathogens, including *V. tasmaniensis* [16], *V. aestuarianus* [17], and *V. coralliilyticus* [18]. *V. coralliilyticus,* long established to be a coral pathogen [19–21], has also been implicated in significant mortality events of larvae at shellfish hatcheries [11, 22–25] and of *C. gigas* larvae in laboratory experiments [18, 24–26]. The outer membrane protein OmpU and the ToxR transcriptional regulator have also been implicated in virulence of *V. coralliilyticus* towards *C. gigas* larvae [27]. However, it is unclear if there are other genetic mechanisms contributing to *V. coralliilyticus* oyster larvae pathogenesis and what their conservation is across the *Vibrio* genus and whether other *Vibrio* strains found within aquaculture settings interact with shellfish and what their impacts on oyster health may be. Genome comparisons of vibrios with varying virulence phenotypes are an imperative step in identifying the genes associated with pathogenesis.

In this study, the genomes of 42 *Vibrio* isolates were sequenced and compared to identify and characterize conserved genomic features contributing to mortalities of *C. gigas* larvae. These isolates, along with nine previously sequenced strains, were tested for pathogenicity towards *C. gigas* larvae. Most isolates exhibiting pathogenic phenotype were identified as belonging to the *V. coralliilyticus* species group, emphasizing the identity of this species as a notable pathogen. By contrasting phenotypes with genotype data, we present an in-depth comparative analysis into the genomic repertoire of pathogenic and non-pathogenic vibrios found in aquaculture environments. Additionally, we introduce insights into the genomic content of *V. coralliilyticus* strains, and put forward evidence for what genes may be responsible for *V. coralliilyticus* vibriosis of *C. gigas* larvae.

## Results

### Pathogenicity of *Vibrio* isolates against *C. gigas* larvae

**Fig. 1:**
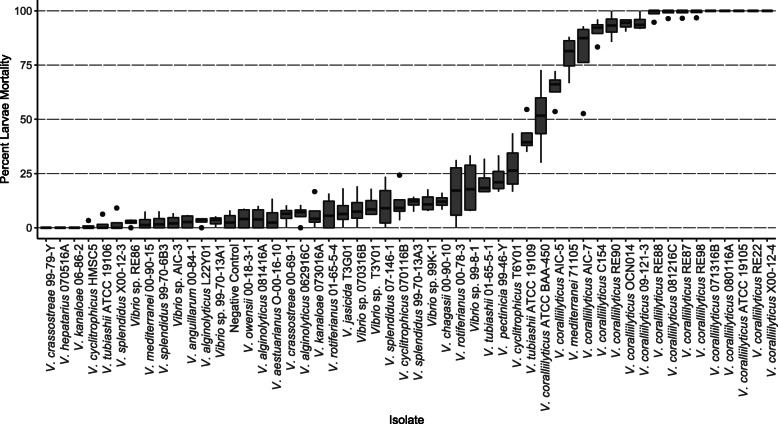
Mortality of *C. gigas* larvae in pathogenicity experiments tested against 51 *Vibrio* strains. Boxes represent the median value, the 25th percentile, and the 75th percentile. Outliers represent values greater than 1.5 times outside these percentiles. Percent mortalities within no-bacterial controls averaged 6.8% (std. dev. 0.077)

Survival of larvae with non-control bacterial strains (*n* = 49) ranged from 0 to 100%, with an average mortality across all trials of 35.7% (std. dev. 40.3%) and median of 13.0% (Fig. 1). Results approximate a bimodal distribution, with individual isolates displaying either high pathogenicity or having little effect on oyster survival. Members of the high-pathogenicity group consistently caused larvae mortalities between 51 and 100% and primarily included isolates identified as *V. coralliilyticus* strains. Only two isolates not classified as *V. coralliilyticus* resulted in high mortalities: strain *Vibrio* sp*.* strain RE88 (99% ± 3%) and *V. mediterranei* strain 71,105 (79% ± 0.10%). The low-pathogenicity group was comprised of a more diverse set of *Vibrio* species. Thirty-nine isolates tested positive for hemolytic activity on 5% horse blood agar and 44 isolates tested positive for proteolytic activity on 2% skim milk agar (Additional file 1: Table S1).

### Genome features, phylogenetics, and comparative genomics

**Fig. 2:**
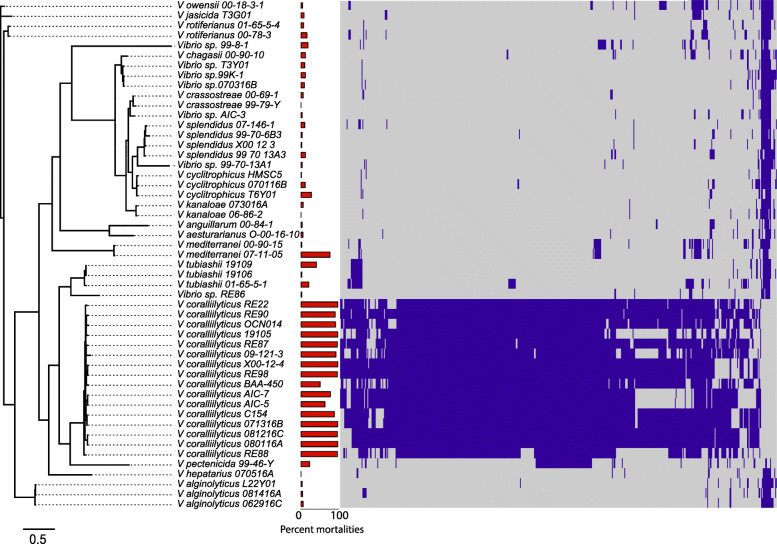
Phylogeny Genes of *Vibrio spp.* associated with oyster pathogenicity. Phylogeny of tested isolates and heatmap of hierarchical clustering of presence and absence of genes significantly correlating (*p* < 0.005) with larval survival in pathogenicity assays. Virulence and phylogeny of *Vibrio spp*. Phylogenomic tree built from 102 universal conserved genes. Bar plot adjacent to each tip label represents the average percent of mortalities of *C. gigas* larvae after 72 h of bacterial challenge. Dark blue indicates presence of a gene and grey indicates absence in the heatmap

To better understand the genomic potential of hatchery-associated vibrios and identify traits that are associated with virulence, we conducted whole-genome shotgun sequencing, assembly, and annotation of the 42 isolates (Table 1). Isolates spanned a wide range of species within the *Vibrio* genus. Ten newly sequenced genomes were designated as *V. coralliilyticus* based on ANI relationships (Additional file 2: Fig. S1). Eight strains did not have > 95% ANI with any reference type strains, preventing species-level classification. One of these, *Vibrio* sp. RE88, was closely related to the *V. coralliilyticus* ATCC BAA-450^T^ at 94% ANI, but under the species-threshold of 95–96% ANI. Three strains, T3Y01, 070316B, and 99 K-1, all had over 98% ANI among pairs, but were not related to any other reference genome in the database tested, indicating that these are possibly a novel species group. A universal gene phylogeny supports the taxonomic assignments based on ANI relationships (Fig. 2), and clusters most of the isolates into two main clades: the Coralliilyticus clade and the Splendidus clade. These data were congruent with a phylogenetic tree built from concatenated sequences of the computationally-determined core genome of 154 Vibrionaceae genomes (Additional file 3: Fig. S2), scoring a Kuhner-Felsenstein (KF) [31] distance of 0.209.

**Table 1:** Bacterial strains and genomes used in this study

Species	Strain	Year	Location	Reference	Accession ID
*V. alginolyticus*	062916C	2016	Oregon	This study*	VTYI00000000
*V. alginolyticus*	081416A	2016	Oregon	This study*	VTYF00000000
*V. alginolyticus*	L22Y01	2015	Washington	This study*	VTYJ00000000
*V. anguillarum*	00–84-1	2000	Washington	This study*	VTYO00000000
*V. chagasii*	00–90-10	2000	Hawaii	This study*	VTXW00000000
*V. coralliilyticus*	071316B	2016	Oregon	This study*	VTYG00000000
*V. coralliilyticus*	080116A	2016	Oregon	[28]	GCA_002286405
*V. coralliilyticus*	081216C	2016	Oregon	This study*	VTYE00000000
*V. coralliilyticus*	09–121-3	Unknown	Unknown	This study*	VTXP00000000
*V. coralliilyticus*	19,105	1965	Conneticut	[22]*	VTXA00000000
*V. coralliilyticus*	AIC-5	2015	New Jersey	This study*	VTXB00000000
*V. coralliilyticus*	AIC-7	2015	New Jersey	[28]	GCA_002287625
*V. coralliilyticus*	BAA-450	1999	Indian Ocean	[20]	ACZN00000000
*V. coralliilyticus*	C154	2017	Washington	This study*	VTYL00000000
*V. coralliilyticus*	OCN014	2010	Palmyra Atoll	[29]	GCA_000763535
*V. coralliilyticus*	RE22	2000	Oregon	[23]	GCA_003391375.1
*V. coralliilyticus*	RE87	1999	Hawaii	[11]	GCA_002286655
*V. coralliilyticus*	RE88	2000	Hawaii	[11]	VTYQ00000000
*V. coralliilyticus*	RE90	2000	Hawaii	[11]	VTYR00000000
*V. coralliilyticus*	RE98	2000	Oregon	[23]	GCA_000772065.1
*V. coralliilyticus*	X00–12-4	2000	Oregon	[23]*	VTYS00000000
*V. crassostreae*	00–69-1	2000	Washington	This study*	VTXY00000000
*V. crassostreae*	99–79-Y	1999	Washington	[23]*	VTXZ00000000
*V. cyclitrophicus*	HMSC5	2000	Oregon	[27]	VTYP00000000
*V. cyclitrophicus*	T6Y01	2015	Washington	This study*	VTYK00000000
*V. cyclitrophicus*	070116B	2016	Oregon	This study*	VTYB00000000
*V. hepatarius*	070516A	2016	Oregon	This study*	VTYD00000000
*V. jasicida*	T3G01	2015	Washington	This study*	VTXQ00000000
*V. kanaloae*	06–86-2	2006	Oregon	[30]	VTXR00000000
*V. kanaloae*	073016A	2016	Oregon	This study*	VTXS00000000
*V. mediterranei*	00–90-15	2000	Hawaii	This study*	VTXV00000000
*V. mediterranei*	71105	Unknown	Florida	This study*	VTXU00000000
*V. owensii*	00–18–3-1	2000	Hawaii	This study*	VTXX00000000
*V. rotiferianus*	00–78-3	2000	Hawaii	This study*	VTYN00000000
*V. rotiferianus*	01–65–5-4	2000	Hawaii	This study*	VTXT00000000
*Vibrio* sp.	070316B	2016	Oregon	This study*	VTYC00000000
*V. pectenicida*	99–46-Y	2000	Washington	This study*	VTXC00000000
*Vibrio* sp.	99–70-13A1	2000	Oregon	This study*	VTXD00000000
*Vibrio* sp.	99–8-1	1999	Washington	This study*	VTXE00000000
*Vibrio* sp.	99 K-1	2000	Hawaii	This study*	VTXF00000000
*Vibrio* sp.	AIC-3	2016	New Jersey	This study*	VTXG00000000
*V. aestuarianus*	O-00-16-10	Unknown	Washington	This study*	VTXH00000000
*Vibrio* sp.	RE86	2000	Hawaii	[11]*	VTXI00000000
*V. splendidus*	T3Y01	2015	Washington	This study*	VTXJ00000000
*V. splendidus*	07–146-1	2000	California	This study*	VTXK00000000
*V. splendidus*	99–70-13A3	2000	Oregon	This study*	VTXL00000000
*V. splendidus*	99–70-6B3	2000	Oregon	This study*	VTXM00000000
*V. splendidus*	X00–12-3	2000	Oregon	This study*	VTXN00000000
*V. tubiashii*	01–65–5-1	2000	Hawaii	This study*	VTXO00000000
*V. tubiashii*	19106	1965	Connecticut	[22]	GCA_000259295
*V. tubiashii*	19109	1965	Connecticut	[22]	GCA_000772105

*Genome sequence and assembled as a part of this work

The core, flex, and pan-genomes of isolate genomes were classified to determine the functional composition of the conserved and unique genes. OrthoMCL [32] clustering defined a total of 1693 core genes, 11,327 flexible genes, and 23,933 genes in the pan genome of sequenced strains. Core and pan-genome gene accumulation plots were created (Additional file 4: Fig. S3), and the curve of the pan-genome plot and the Heaps law model (alpha = 0.47) indicated an open-pan genome amongst these isolates.

The observed distribution of functional categories of the genes significantly correlated with the results of larval pathogenicity assays was significantly different than that of the genes in the core genome (chi-square = 3979.2, df = 19, *p* < 0.001). The functional categories “Carbohydrate transport and metabolism”, “Transcription”, “Secondary metabolites biosynthesis, transport and catabolism”, “Function unknown”, and “Signal transduction mechanisms” were significantly enriched in the flexible genome relative to the core genome (*p* < 0.05). The category of genes without any significant hits to the GAMMAPROTNOG database was also significantly enriched in the flex genome (*p* < 0.05).

### Genes associated with *C. gigas* larvae pathogenicity

To identify genes associated with *C. gigas* larval mortalities we implemented a phylogenetic logistic regression model using gene presence/absence and pathogenicity assay data for each isolate. A total of 508 genes were identified to have a significant negative correlation with larvae survival (*p* < 0.005), while only 19 genes had a significant positive correlation with survival (Fig. 2 and Additional file 5: Table S2). Since most of the pathogenic isolates were putatively assigned as *V. coralliilyticus* species, we used coding sequences from the complete genome sequence of *V. coralliilyticus* strain RE22 (Accession: GCA_003391375) as a reference for gene annotations, and to compare the synteny of contigs from isolate genome assemblies. Three eggNOG functional categories were significantly enriched in the group of genes correlated with high mortality/low survival phenotypes compared to the functional composition of core genome from all strains (Supporting Fig. 3): “Carbohydrate transport and metabolism” (G; *p* = 0.0365); “Secondary metabolite biosynthesis, transport and catabolism” (Q; *p* = 5.27E-06); “Function unknown” (S; *p* = 4.11E-11); and a fourth category containing genes that did not have significant hits to the database (*p* = 0). Metabolic genes were a dominant component of these functional categories and included chitinase A precursors, a bile acid symporter, a pectate lyase, and multiple sugar degradation enzymes (Additional file 6: Table S3).

Additional genes found to be negatively correlated with larvae survival included those with annotations putatively involved with host virulence. A group of *reb* genes were significant (C = 3.48, *p* = 0.0005) (Table 2), with most being exclusive to and conserved within the *V. coralliilyticus* group of isolates. The putative *reb* genes were located within a larger putative genomic island of the RE22 complete genome (~ 30 KB) that encoded multiple genes found to be significant with larvae pathogenicity outcomes, including an endolysin (AXN33720.1), a methyltransferase (AXN33721.1), a putative methyl-accepting chemotaxis protein (AXN33724.1), and multiple hypothetical proteins.

**Table 2:** Features of the putative Reb-coding region of *Vibrio coralliilyticus* strain RE22

*V. coralliilyticus* RE22 coding sequence accession^a^	Amino Acid Length	Phylogenetic Logistic Regression *p*-value	Putative function	Domain Hit^b^	Domain Accession
AXN33720.1	296	**5.73E-04**	Muraidase	Muraidase	pfam11860
AXN33721.1	261	**5.02E-04**	SAM-dependent methyltransferase	Methyltransf 23	pfam13489
AXN33722.1	518	**5.02E-04**	Cystine-binding periplasmic protein	Periplasmic Binding Protein Type 2	cl21456
AXN33723.1	207	**5.02E-04**	HisM	HisM	cl34036
AXN33724.1	692	**5.02E-04**	MCP protein	PDC2 MCP like	cd12912
AXN33725.1	258	**5.02E-04**	Intermediate filament tail domain protein	Lamin Tail Domains	pfam00932
AXN33726.1	320	**5.02E-04**	hypothetical protein	ND	ND
AXN33727.1	140	**5.02E-04**	hypothetical protein	ND	ND
AXN33728.1	99	**5.02E-04**	hypothetical protein	ND	ND
AXN33729.1	261	**5.02E-04**	hypothetical protein	ND	ND
AXN33730.1	235	**5.02E-04**	hypothetical protein	ND	ND
AXN33731.1	283	**5.02E-04**	hypothetical protein	ND	ND
AXN33732.1	197	**5.02E-04**	hypothetical protein	ND	ND
AXN33733.1	304	0.9998	short chain dehydrogenase	sepiapterin reductase	TIGR01500
AXN33734.1	658	0.99998	hypothetical protein	ND	ND
AXN33735.1	632	1	phospholipase	PLDc SF	cl15239
AXN33736.1	420	1	Tat pathway signal protein	ND	ND
AXN34531.1	184	1	hypothetical protein	ND	ND
AXN33737.1	297	ND	hypothetical protein	ND	ND
AXN33738.1	297	ND	hypothetical protein	ND	ND
AXN33739.1	1187	ND	hypothetical protein	ND	ND
AXN33740.1	88	**5.02E-04**	hypothetical protein	ND	ND
AXN33741.1	63	**5.02E-04**	Reb-like protein	Reb	cl13232
AXN33742.1	90	**5.02E-04**	Reb	Reb	pfam11747
AXN33743.1	87	**5.02E-04**	hypothetical protein	conj TIGR03752	cl26990
AXN33744.1	142	**5.02E-04**	Reb-like protein	Reb	cl13232
AXN33745.1	97	**3.45E-03**	hypothetical protein	ND	ND
AXN33746.1	87	**5.02E-04**	Reb	Reb	pfam11747
AXN33747.1	86	**5.02E-04**	Reb	Reb	pfam11747
AXN33748.1	88	**5.02E-04**	Reb	Reb	pfam11747
AXN33749.1	66	**5.02E-04**	Reb-like protein	Reb	cl13232
AXN33750.1	403	**5.02E-04**	AraC transcriptional reg.	AtoC	cl34427

*ND* No domain hits were found for that sequence.

Bold values indicate significant phylogenetic logistic regression results (*p* < 0.005).

^a^ Accession number GCA_003391375.1

^b^ Top domain identified using the Conserved Domain Database

Genes coding for a Type 1 Secretion System (T1SS) (AXN34790.1, AXN34851.1, and AXN34791.1) were significant based on the phylogenetic logistic regression analysis (*p* < 0.0005), although this region, located in *V. coralliilyticus* strain RE22 on the p337 plasmid, was not conserved in all isolates of this species. Additional genetic features with potential virulence functions included a coding sequence with a haem-degrading domain (AXN33843.1; C = 3.48, *p* = 0.0005), a gene with pore-forming and peptidase domains (AXN34842.1), regulatory proteins and hypothetical proteins (C > 2.8, *p* < 0.005), a gene coding for a necrosis-inducing protein NPP1 (AXN30583.1; C = 3.48, *p* = 0.0005), and a gene with a domain similar to the cysteine proteinase peptidase_C58 superfamily (AXN33813.1; C = 5.21, *p* = 0.0005).

**Fig. 3:**
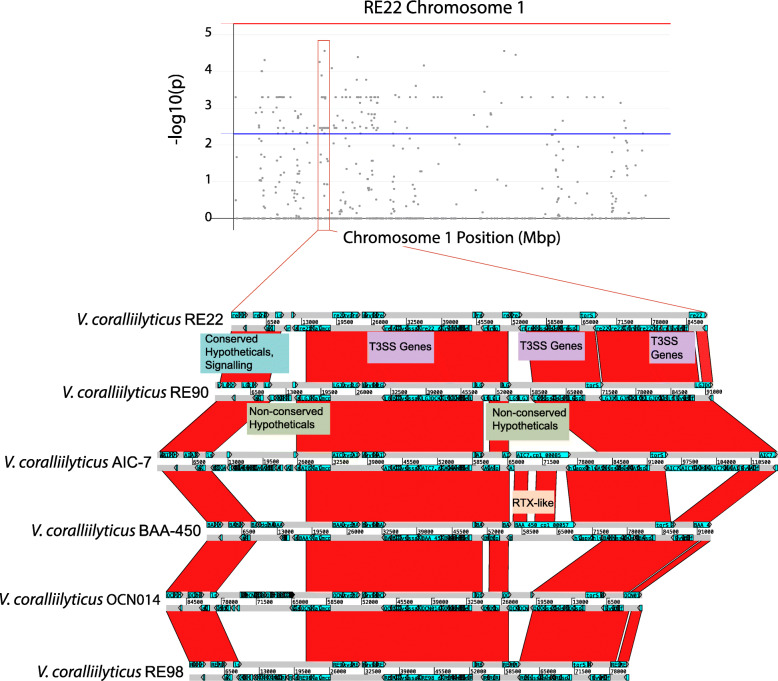
Manhattan Plot with *P*-value results for *Vibrio* genes correlating with larvae mortalities and ACT plots of the CPI-1 region of six *V. coralliilyticus* strains. Manhattan plot (top) shows *p*-values for genes on the Chromosome 1 of *V. coralliilyticus* RE22 that were tested with a phylogenetic logistic regression for correlation with larvae mortalities. *P*-values are log-transformed. Blue line corresponds to a significance cut-off of *p* < 0.005. CPI-1 regions of genomes (bottom) were aligned using BLASTN and results for each pairwise alignment were visualized in Artemis Comparison Tool (ACT) in order to visually compare gene synteny and sequence similarity between CPI-1 regions of *V. coralliilyticus* strains

Within the large chromosome of the RE22 genome, a number of syntenic loci whose presences were significantly negatively correlated with larvae survival had annotations for a functional Type 3 Secretion System (T3SS; Fig. 3). The genomic region containing these genes corresponds to the pathogenicity island CPI-1 [33]. In addition to encoding a T3SS and effectors, this region includes genes for multiple transcriptional regulators, a rhombotail lipoprotein, an anthranilate synthase component II, the hemolysin locus *vchAB*, a glutathione-dependent formaldehyde-activating enzyme (GFA)-related enzyme, and hypothetical proteins (Additional file 7: Fig. S4). This genomic island is conserved in all *V. coralliilyticus* genomes examined in this study, as well as the closely-related *Vibrio* sp*.* strain RE88. It is not encoded in *V. mediterranei* strain 71,105, though, which was found to cause high mortalities in oyster larvae. The CPI-1 island was not fully present in strains causing low mortality rates. *V. pectenicida* strain 99–46-Y, which causes low levels of mortality, encodes a partial CPI-1-like island, which encodes the T3SS structural genes and putative effectors (Additional file 8: Fig. S5).

A comparison of the CPI-1 region of six *V. coralliilyticus* genomes revealed that the general architecture of this region ranged from 81 to 112 kb in length and consisted of several regions of conserved gene content interspaced with more variable regions (Fig. 3), including a locus containing an RTX-like coding sequence and T1SS machinery. Phylogenetic analysis of the *sctV* gene, which encodes a conserved protein of the T3SS, indicated that this gene is more similar to those of species of *Yersinia*, *Salmonella*, *Escherichia*, *Edwardsiella* and *Shewanella*, and separate from the *sctV* genes of *V. parahaemolyticus*, *V. cholerae*, *V. alginolyticus*, and *V. harveyi* (Additional file 9: Fig. S6).

## Discussion

Here, we examined the ability of *Vibrio* isolates to cause mortality of *C. gigas* larvae under controlled experiments. Many of these isolates were collected from hatcheries where *C. gigas* larvae are produced. Comparative analysis of the genomes of these 51 isolates identified genes specifically associated with a pathogenic lifestyle from the large pan-genome. We found a strong phylogenetic signal among the strains that caused high mortalities; 15 out of 17 strains tested resulting in 50% or higher mortality rates were putatively identified as *V. coralliilyticus* strains, and one, *Vibrio* sp. RE88, was found to be the closest outgroup to this species. Strain *V. mediterranei* 71,105, was the lone pathogen not found within this clade. *V. coralliilyticus* has been previously implicated in disease outbreaks at a West Coast hatchery [11]. Importantly, we did not have a *V. coralliilyticus* strain that did not cause mortalities in tests with *C. gigas* larvae, so although we attempted to control for phylogenetic signal with the phylolm package, we cannot definitively assert that the genes detected with this model are directly related to pathogenesis rather than just being a trait of the species serving some other ecological function. Still, in this discussion we highlight gene features, many of which are conserved in the *V. coralliilyticus* strains we tested, significantly correlated with mortalities, signifying that these genes may set apart pathogens from other vibrios ubiquitous in hatchery and marine environments.

A number of gene clusters negatively correlated with oyster survival were homologous to genes of the CPI-1 pathogenicity island of *V. coralliilyticus* ATCC BAA-450 [33]. Pathogenicity islands are a common way for bacteria to distribute large sets of virulence genes via horizontal gene transfer [34]. The most conspicuous virulence factor encoded by the CPI-1 is a putative T3SS. T3SSs are often used by pathogenic bacteria to invade or manipulate host cells. For example, the T3SS-2 of *Vibrio parahaemolyticus* facilitates host cell invasion [35]. The observation that the conserved T3SS gene, *sctV*, is more similar to non-Vibrio T3SS genes is consistent with a previous phylogenetic analysis [36] and suggests that the T3SS, and perhaps the surrounding the CPI-1 island, was acquired through horizontal gene transfer.

It is important to note that, although *V. pectenicida* 99–46-Y appears to encode a relatively complete CPI-1, it was not virulent in our testing. This allows for a comparison of the gene content of the CPI-1 from pathogens with that of the CPI-1 of *V. pectenicida* 99–46-Y in order to identify those genes that may be specific to oyster larvae pathogenesis. Genes located in CPI-1 of *C. gigas* larvae pathogens, but absent in the non-pathogenic *V. pectenicida* 99–46-Y, included those annotated as the *vchA* hemolysin (AXN30429.1) and another secreted lysin, *vchB* (AXN30428.1). These genes are highly similar to two homologs of *V. vulnificus* (*vvhA* [100% coverage and 76.86% identity], *vvhB* [100% coverage and 59.52% identity]), which are known to cause epithelial damage and contribute to intestinal growth of the bacterium [37]. Additionally, VchA of *V. coralliilyticus*, was shown to lyse eukaryotic cells, including erythrocytes [38]. Interestingly, though, culture supernatants from strains of recombinant *V. cholerae* expressing the *vchA* gene did not cause significant mortality to *C. gigas* larvae [18], indicating that although there appears to be a role for this protein in lysing eukaryotic cells, its role as an independent virulence factor in oysters is unclear.

As filter feeding organisms, oysters inherently accumulate large amounts of bacteria from the surrounding water, leading to many coincidental interactions with pathogens. Like other invertebrates, oysters rely on innate immunity and phagocytic cells (e.g., hemocytes in oysters) to protect against pathogenic microbes. Hemocytes are circulatory cells that develop by the early D-veliger larvae stage (17 h post-fertilization) [39] and eliminate bacterial pathogens through specific binding mechanisms, phagocytosis, and subsequent digestion mechanisms [40–43]. Exposure of *C. gigas* larvae to pathogenic *V. coralliilyticus* is known to cause a decline in feeding rate, an activation of the immune response (e.g., hematopoiesis, activation of non-self recognition mechanisms, and production of antimicrobial peptides), and modulation of cell membrane composition [26]. Less is known, though, about the strategies that pathogens employ to escape the immune system of Pacific Oyster. Interestingly, *V. coralliilyticus* RE22 has been shown to suppress immune signalling pathways of Eastern Oyster (*Crassostrea virginica*) larvae [44]. Our work expands on these findings by identifying additional conserved mechanisms that *Vibrio* pathogens may utilize to circumvent immune responses by oyster larvae.

For instance, multiple genes of a ~ 30 Kb-long locus conserved within the genomes of the tested *V. coralliilyticus* isolates and *Vibrio* sp. strain RE88 were found to be negatively correlated with larvae survival. This locus includes multiple *reb* genes and their nearby coding sequences, which are predicted to encode “R-bodies”. R-bodies are insoluble bacterial proteins that confer a phenotype known as the “killing trait” [45]. R-bodies of the intracellular symbiont, *Caedibacter*, switch between two stable conformations in response to a stimulus such as an extracellular pH change that occurs during phagocytosis. R-bodies of *Caedibacter* cells, which have been internalized within the lysosomes of a symbiont-free competitor paramecium, will then switch into a needle conformation and rupture the bacterial cell wall, whereupon the cytosolic contents will release unidentified toxins that induce paramecium death [45, 46]. Recombinant Reb proteins from *V. nigripulchritudo*, a shrimp pathogen, have been visualized by transmission electron micrographs [47], and the *reb* gene cluster of *V. coralliilyticus* are proposed to be derived from a horizontal gene transfer event [48], although no known role has been identified in this species. This region of genes appears to be highly conserved in *V. coralliilyticus* as it is also present in several *V. coralliilyticus* genomes not included in our study (data not shown). The high correlation of putative *reb* genes with pathogenicity make this region a target for further investigation to determine its role in virulence or survival within the host, whereby *V. coralliilyticus* infections may be facilitated by invading and/or modulating the cells of the oyster immune system.

A locus including gene AXN30831.1 was negatively correlated with high rates of larval survival and was annotated to degrade myo-inositol to acetyl-CoA. *V. coralliilyticus* strain BAA-450 is known to utilize myo-inositol as a carbon source [20]. Pathogens may be encountering inositol in the host environment. For example, host inositol promotes the growth and virulence of *Legionella pneumophila* within amoeba and host macrophages [49]. Phosphatidylinositols, membrane lipids with a myo-inositol sugar in the headgroup, have been found to be important in *C. gigas* growth [50]. Furthermore, there is evidence that both phosphatidylinositol and soluble inositol phosphate signalling plays a role in immune response by bivalve hemocytes [42, 51, 52], and that *C. gigas* larvae challenged with *Vibrio* pathogens show increased in phosphatidyliniositol content [26]. These findings suggest that pathogenic vibrios may modulate oyster immunity by interfering with inositol phosphate signalling, and/or may be able to use host-derived myo-inositol as a carbon and energy source.

Multiple genes annotated to the COG category for carbohydrate utilization were negatively correlated with high larval survival. Two of these were putatively annotated as chitinase genes. One of these is a *chiA* homolog (AXN33250.1) that is uniquely encoded within *V. coralliilyticus* genomes, and is divergent from another *chiA* homolog found to be conserved among the genomes of all *Vibrio* isolates tested. A study by Lin et al. [53] noted that the former homolog was likely introduced into the *V. coralliilyticus* clade via horizontal gene transfer, while the more conserved homolog was hypothesized to be vertically inherited within the genus and under strong purifying selection. Another gene, AXN29974.1, with a partial *N*- acetylglucosamine-binding protein A domain was also negatively correlated with high larval survival. This domain functions in chitin-binding and in *V. cholerae* [54].

Genes for chitin binding and degradation are present in almost all *Vibrio* genomes [53, 55] and chitin metabolism is an important and highly conserved feature, with roles in survival with marine hosts [56], nutrient acquisition [57], and conjugation of extracellular DNA [58]. With regard to oyster hosts, chitin is one of several essential components of their shell matrix [59], and chitin synthase is highly expressed in early stages of *C. gigas* larval development [60], and in hemocytes and the mantle of adults [60, 61]. Interestingly, growth of *V. coralliilyticus* strain S2052 on chitin not only induces expression of chitin utilization genes, but also genes related to host colonization, pathogenesis (including *reb-*type genes), and natural competence [62]. Our observation of a correlation of these putative chitin utilization genes with pathogenicity supports a hypothesis that chitin is a key mediator of interactions between oysters and *Vibrio* pathogens.

Multiple genes with putative functions for nutrient acquisition were found to be significantly correlated with pathogenicity. Several were proteases, which can aid in colonization of and persistence within a host, degradation of host biomass for energy and carbon acquisition, or the breakdown of accumulated waste [63]. One of these, a S8 serine peptidase (AXN34464.1) was highly similar to a protein (97.54% identity) found in the secretome of a *V. coralliilyticus* P1 *vcpA* mutant that is virulent towards *Artemia* and *Symbiodinium* [64]. Additional putative metabolic functions found to correlate negatively (Additional file 6: Table S3) with larval survival included a conserved locus spanning three operons with genes coding for disaccharide transport, as well as the high-affinity *pstSCAB* phosphate transporter. These metabolism features correlated with pathogenicity may reveal strategies and adaptations for colonization of a host or other environments.

Genes *vcpA*, *vcpR*, *toxR* were not found to be significantly associated with pathogenicity, despite being known virulence factors of *V. coralliilyticus* [18, 27, 65]. These genes were generally found to be conserved among all genomes tested here, precluding detection of statistical relationships between their presence/absence and virulence. Additionally, multiple known virulence genes of *V. splendidus* pathogens shown to be important for virulence of species were not significantly correlated with our pathogenicity results, including. Type 6 Secretion Systems (T6SS) [14, 15, 66]. Apparent differences between previous results and ours may arise from minor, but important, differences in sequences of each locus, differences in methods applied for gene clustering, environmental conditions such as temperature, or divergent controls of transcription of conserved genes between pathogenic and non-pathogenic isolates. Host factors such as age (larvae versus adult *C. gigas*) or a host genotype factor [67] may also account for the divergent results. An additional caveat of our work is that gene clusters detected as in-paralogs, such as one of the T6SS clusters present in many genomes of pathogens, were not analyzed with the phylogenetic logistic regression precluding direct comparisons with previous results.

While only 19 homologous gene clusters were found to be positively correlated with survival of larvae, 508 homologs were identified to negatively correlate with high survival rates, indicating that *Vibrio* pathogenesis towards *C. gigas* larvae is a complex trait involving multiple adaptive features. The wide diversity of functions encoded by genes correlated with larval pathogenicity, included ones with more classical virulence functions such as proteases and T3SS-related functions, but also genes putatively encoding functions for colonization, nutrient acquisition, and those with unknown functions. Our results indicate that infection of oyster larvae by *V. coralliilyticus* may involve an intracellular stage facilitated by the T3SS and the Reb defense system. We therefore hypothesize that these pathogens interact with the immune system of larvae in a similar manner that is seen for *V. coralliilyticus* infection of the coral *Pocillopora damicornis* and of *V. tasmaniensis* with oysters, where infection proceeds through the formation of intracellular bacterial aggregates, followed by the repression of host innate immunity, host cell lysis and extensive tissue damage [15, 68, 69]. The genetic features described here will be fruitful targets for future mechanistic studies of the oyster larvae pathogenesis.

## Conclusions

This work highlights the importance of *Vibrio* genes associated with Pacific oyster (*Crassostrea gigas*) larvae mortalities. Forty-two newly sequenced hatchery-associated *Vibrio* genomes are presented in this study. Out of a wide range of *Vibrio* species, the most prevalent pathogenic species identified were *V. coralliilyticus* strains. Important considerations of our work are that both pathogenic and non-pathogenic vibrios were isolated from hatcheries when no disease symptoms were evident, and that vibrosis in oysters does not result from one single etiological agent or genetic feature, instead appearing to be a multifaceted trait enabled by many loci. These findings point to the complexity of vibrosis outbreaks, whereby environment, microbial community structure, and genotypic and phenotypic features of *Vibrio* populations all have important roles in disease outcomes. These findings have meaningful implications on monitoring and management practices of aquaculture, where multiple genetic traits and species are all relevant and must be considered with regard to controlling disease.

## Methods

### Pathogenicity of *Vibrio* isolates against *C. gigas* larvae

*C. gigas* larvae were collected 48 h post-spawn from a hatchery in Newport, Oregon. Seawater was collected from an onsite intake line and autoclaved. *Isochrysis sp.* was used for larvae food. *Vibrio* isolate strains were grown at a temperature of 25 °C (with the exception of strain 99–70-13A1, which was grown at 23 °C) in LB + 3% NaCl (LBS) to an OD600 of 0.8. Cell cultures were serially diluted in sterile seawater, and cell densities were confirmed by colony forming units (CFUs) on LBS. On the day of larvae collection, larval density was estimated by microscopy. The absence of *Vibrio* bacteria in unamended stocks of *C. gigas* larvae and algal feed was confirmed by direct plating on Thiosulfate Citrate Bile Salts Sucrose (TBCS) agar.

Challenges with *Vibrio* isolate strains and *C. gigas* larvae were performed in sterile 24-well plates. For each replicate challenge, 20–30 larvae and 10^5^ *Vibrio* cells/ml in sterile seawater were added to each well at a final volume of 6 ml. Bacteria-free controls were performed with larvae alone in sterile seawater. Known pathogenic (*V. coralliilyticus str.* RE98) and non-pathogenic (*V. cyclitrophicus* HMSC5) isolates, added at d 10^5^ *Vibrio* cells/ml in sterile seawater, were used in challenges as positive and negative controls, respectively. Wells were sealed with parafilm and covered during subsequent incubations at 25 °C for 48 h. The neutral red uptake assay was performed post-challenge to quantify larvae mortality. Briefly, neutral red solution was added to each well at a final concentration of 0.0002% (v/v). After 3 h, 30 μl of 10% buffered formalin was added to wells. Larvae were visualized with a dissecting microscope to characterize morphology and quantify dye uptake. Each *Vibrio* isolate was tested in quadruplicate, and mean mortality rates and standard deviations are reported. Each strain was tested for hemolytic and proteolytic activity. To test for extracellular proteolytic activity, strains were streaked on LB supplemented with NaCl (3%) and skim milk (2%) agar and incubated at room temperature (~ 21 °C) and 28 °C for 48 h. To test for hemolytic activity, strains were streaked on Nutrient Agar supplemented with horse blood (5%) at room temperature for 48 h.

### Genome sequencing and assembly

All *Vibrio* strains were obtained from culture collections or isolated directly from samples collected from commercial shellfish hatcheries. Isolates from hatcheries were obtained by plating samples of intake water and larvae tanks on TCBS. Colonies characteristic of *Vibrio* spp*.* were restreaked for isolation before growth in liquid broth and cryogenic preservation at − 80 °C.

Genomic DNA from overnight cultures of isolates was purified using the phenol-chloroform extraction method [70] or a DNeasy Blood & Tissue Kits (QIAGEN, Venlo, Netherlands). Quality and quantity of isolated genomic DNA was assessed by electrophoresis on a 0.9% agarose gel, by measuring absorbance wavelengths at 260 and 280 nm, and Qubit fluorometry (Thermo Fisher Scientific, Waltham, MA USA). DNA sequencing libraries of genomic DNAs were prepared using Nextera XT kit (Illumina Inc., San Diego, CA USA). Libraries were pooled together at equal concentrations and sequenced using the Illumina Miseq Platform (2 × 250 bp) at Oregon State University’s Center for Genome Research and Biocomputing (CGRB). Raw sequencing reads were demultiplexed and barcodes removed prior to quality-filtering and trimming using Sickle. High quality reads were then randomly subsampled to obtain approximately 50-fold coverage of the genome and assembled using IDBA-UD version 1.0.6 [71] with iterative k-mer assembly between a 45 and 105 with 4-mer increments. Annotation of contigs > 500 bp was conducted with Prokka with default settings [72]. Completeness and purity of assembled genomes was assessed with the CheckM tool [73]. Average-nucleotide identity (ANI) values between all sequenced genomes and a downloaded set of reference genomes from all *Vibrio* species available from NCBI Genbank (date accessed: May 1st 2018) were calculated using the tool FastANI [74]. A 95% ANI cut-off ratio to reference genomes was used to putatively assign species labels to each of the newly sequenced genomes [74–76].

### Comparative genomics and phylogenomics

Homologous clusters of gene families were identified using the GET_HOMOLOGUES software package [77] with OrthoMCL clustering [32] using default parameters. Orthologous clusters containing two or more members from the same genome (defined as inparalogs) were excluded (*n* = 1087) from subsequent statistical tests. The “core genome” was defined as those genes present in all 51 strains tested, while the “soft core genome” was defined as genes found in greater than 95% of the 51 genomes analyzed. The “flex genome” was defined as those genes present in 5–95% of the genomes. The Heaps law model [78] was used with the function “heaps” in the R package micropan [79] to estimate pan genome openness.

A phylogeny based on single-copy universal genes was built for the 51 tested *Vibrio* genomes using the “bcgTree” pipeline [80]. Briefly, the hmmer program (version 3.1b2, http://hmmer.org/) searched all predicted protein coding sequences from the *Vibrio* genomes against a database of hidden markov model (hmm) profiles of genes present in over 95% of bacterial genomes [81]. Best matches above a gene-specific cut-off were retained and query sequences with the same database match were aligned using MUSCLE with default settings [82]. Resulting alignments were filtered with Gblocks [83] and concatenated with AMAS [84]. RAxML was used to build a partitioned maximum likelihood phylogenetic tree from this alignment, using a GAMMA distribution of rate heterogeneity and individually estimated models of evolution for each gene partition of the concatenated alignment [85].

A phylogeny was constructed from the core genome of all newly sequenced Vibrio genomes, 98 reference *Vibrio* genomes, and the *Enterovibrio norvegicus* DSM 15893 and *Photobacterium gaetbulicola* Gung47 genomes as outgroups. All reference genomes downloaded as nucleotide fastas from RefSeq and annotated using the PROKKA pipeline for consistency. Genomes with an N90 under 10,000 bp, estimated to be less than 90% complete, or with high strain heterogeneity were excluded from further analysis. To define the core genome of this dataset amino acid sequences from all genes were used as input for the GET_HOMOLOGUES pipeline. OrthoMCL was used to cluster protein sequences with 50% minimum coverage, 50% minimum identity and a default e-value of 0.00001. A concatenated alignment and phylogenomic tree was then created from the core set of homologous genes as described above for the single copy, universal gene data set. The two phylogenies were then analyzed for congruency with one another using the ‘treedist’ function in the package ‘phangorn’ [86] with KF distance.

### Detection of gene orthologs correlated with pathogenicity

To identify the set of genes in *Vibrio* genomes significantly associated with larvae mortalities, correlations between presence or absence of genes and the results of larval pathogenicity assays were calculated with a phylogenetic logistic regression model of the R package “phylolm” (86). Gene presence/absence across all genomes was considered binary response variable and percent survival was considered a predictor variable for each model, and all single-copy genes present in 3 or more genomes but not more than 46 genomes (*n* = 6676) were tested. The phylogeny constructed from single-copy, universal genes was used as input for the model. Gene occurrences were considered significantly correlated with larvae data using a *p*-value cutoff of 0.005, and the effect of the relationship was determined by the strength and sign of the correlation estimate.

### Functional category analysis

Orthologous gene clusters were functionally annotated against the Non-supervised Orthologous Groups database (eggNOG) [87]. Each orthologous cluster was aligned with MUSCLE [82], and an hmm profile was built HMMER, which was then used to search against the eggNOG GAMMAPROTNOG database to provide a consistent annotation of each respective cluster. A chi-squared test was conducted to compare the distribution of functional categories for genes identified to be significantly negatively correlated with larvae survival to the distribution of functional categories for genes in the core genome. Pairwise post-hoc tests for each functional category were then performed with an FDR correction to determine which individual functional categories were significantly different between each dataset. Categories were considered enriched at a significance level of *p* < 0.05.

### Phylogeny of T3SS genes

To infer phylogenetic relationships of specific genes, including the *sctN* (the Type III secretion cytoplasmic ATPase) and *sctV* (Type III secretion inner membrane protein) homologues, hmm profiles for genes of interest were obtained from the Pfam database [88]. These profiles were used to search against a database of putative protein sequences from the newly sequenced and reference *Vibrio* genomes. Best matches falling within an inclusion threshold of an E-value ≤0.01 were selected as putative homologues for downstream analyses. A multiple sequence alignment with identified homologues, as well as a database of reference proteins for sequences of interest, was created with MUSCLE [82]. A maximum-likelihood phylogenetic tree was constructed using RAxML with GAMMA model of rate heterogeneity and the LG model of protein substitution and empirical base frequencies (as determined by automated protein model assignment).

## Supplementary information

**Additional file 1 Table S1**. Hemolysis and proteolysis reactions.

**Additional file 2 Fig. S1.** Heatmap and dendrogram of Average Nucleotide Identity (ANI) calculations between pairs of different Vibrio strains, including strains used in this study and reference strains. ANI of ≥95% with a Type Strain of a species was used for species designation of newly sequenced strains. Strains without ANI ≥ 95% with any Type Strain were designated “sp.”

**Additional file 3 Fig. S2.** Phylogenetic Tree of strains used in this study and reference strains. A concatenated alignment of 686 conserved amino acid sequences from single copy orthologous genes was used to construct a maximum likelyhood phylogeny.

**Additional file 4 Fig. S3.** Core and Pan-genome of 51 isolates in this study. (A) the core-genome represents all genes shared by each genome added. (B) The pan-genome represents the accumulation of all genes among all genomes with the addition of each genome.

**Additional file 5 Table S2.** Phylogenetic logistic regression results.

**Additional file 6 Table S3.** Metabolism and nutrient acquisition genes significantly correlated with larvae survival/mortality.

**Additional file 7 Fig. S4.** Count distributions of bactNOG functional categories of genes among categories of different gene sets. The “Core in All” column refers to the core genome (*n* = 1693 gene clusters) of all strains (*n* = 51). The “Core in Non-Pathogens” column refers to the core genome (*n* = 1775 gene clusters) of all strains (*n* = 34) that are non-pathogenic (0.5 or lower mortality ratio). The “Core in Pathogens” column refers to the core genome (*n* = 2646 gene clusters) of pathogenic strains. The “Exclusive to Non-Pathogens” column refers to genes (*n* = 4010 gene clusters) that are found only in non-pathogens, but not necessarily conserved. The “Exclusive to Pathogens” column refers to genes (*n* = 1981 gene clusters) that are found only in pathogens, but not necessarily conserved. The “Flexible” column refers to genes (*n* = 3345 gene clusters) that are found at least once in both pathogens and non-pathogens, but are not conserved in either. The “Phyloglm Significant with Mortalities” column refers to all genes (*n* = 509 gene clusters) that had a significant correlation with pathogenicity towards *C. gigas* larvae.

**Additional file 8 Fig. S5.** Gene organization of the CPI-1 region within the complete genome of *V. coralliilyticus* RE22 and the draft genome of *V. pectenicida* 99–46-Y. Pink arrows indicate that the gene had a negative correlation with larval survival in virulence assays. Blue arrows indicate that there was no significant correlation with survival or that the gene was not tested. Yellow slashed lines indicate contig breaks on the CPI-1 of the *V. pectenicida* strain 99–46-Y draft genome.

**Additional file 9 Fig. S6.** Predicted phylogeny of Type 3 Secretion Systems using SctV amino acid sequences. Clades are color coded. Included in the phylogeny are proteins from tested isolates, specific Vibrio reference genomes, and from COG4789 comprised of more diverse reference genomes. Scale bar represents one change per amino acid. Tree was visualized with iTOL.

**Additional file 10 Table S4.** Pan-genome table (excluding genes only found once in a genome) with strain RE22 for reference loci.

## Data Availability

The draft genomes of the 42 *Vibrio* isolates have been deposited in the GenBank database under project accession no. PRJNA563078.

## Abbreviations

ANI: Average nucleotide identity
eggNOG: Non-supervised orthologous groups database
COG: Clusters of orthologous groups
CPI-1: Coralliilyticus pathogenicity island 1
KF: Kuhner-felsenstein
MARTX: Multifunctional-autoprocessing repeats-in-toxin
RTX: Repeats-in-toxin
T1SS: Type 1 secretion system
T3SS: Type 3 secretion system
T6SS: Type 6 secretion system
