# Supplementary figures and images for: Comparative genomic analysis of Vibrios yields insights into genes associated with virulence towards C. gigas larvae
Source: BMC Genomics. 2020 Aug 31;21:599. doi: 10.1186/s12864-020-06980-6 (PMC7457808; doi:10.1186/s12864-020-06980-6)

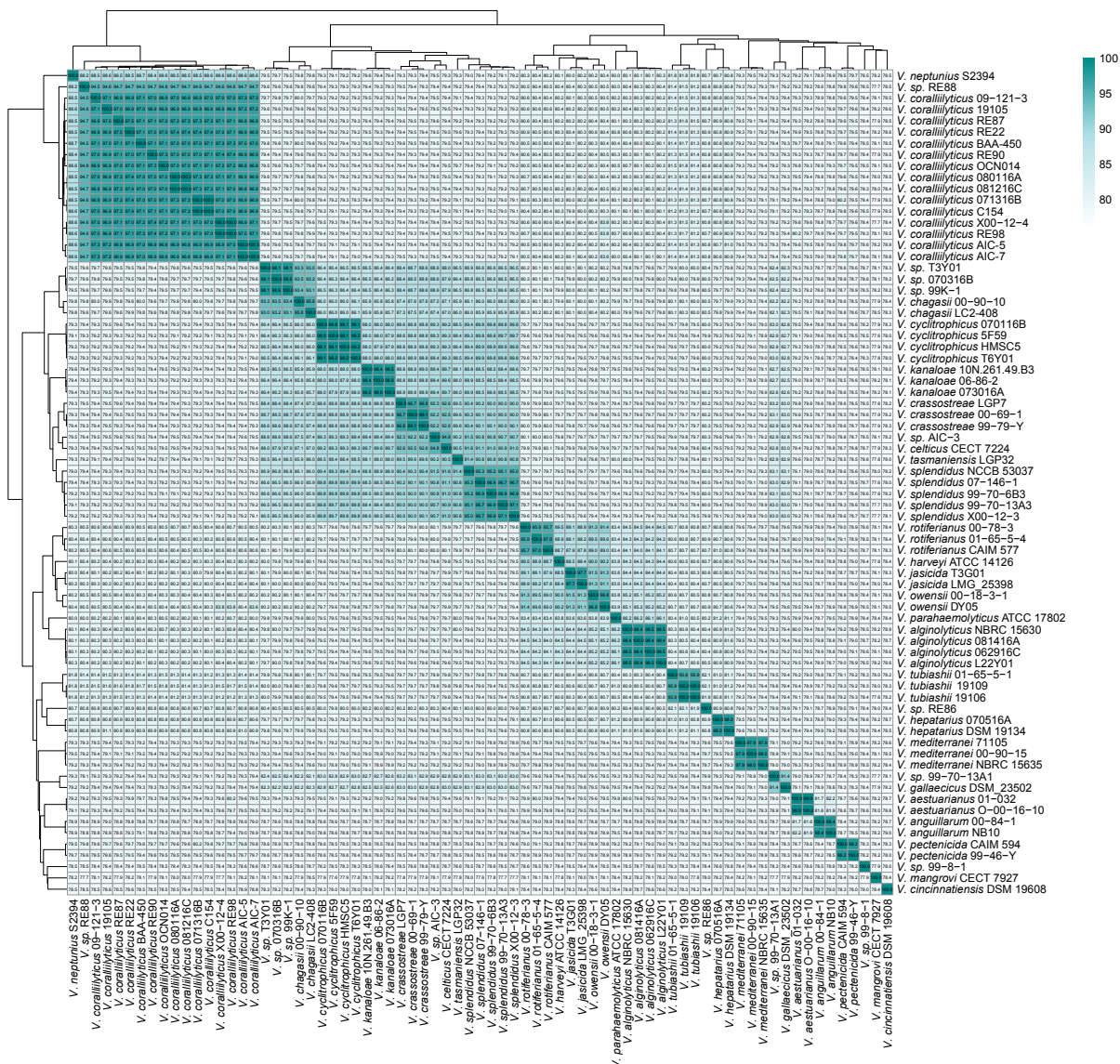

Supplement: Supplementary file 2 — Additional file 2 Fig. S1. Heatmap and dendrogram of Average Nucleotide Identity (ANI) calculations between pairs of different Vibrio strains, including strains used in this study and reference strains. ANI of ≥95% with a Type Strain of a species was used for species designation of newly sequenced strains. Strains without ANI ≥ 95% with any Type Strain were designated “sp.” [file 12864_2020_6980_MOESM2_ESM.pdf]

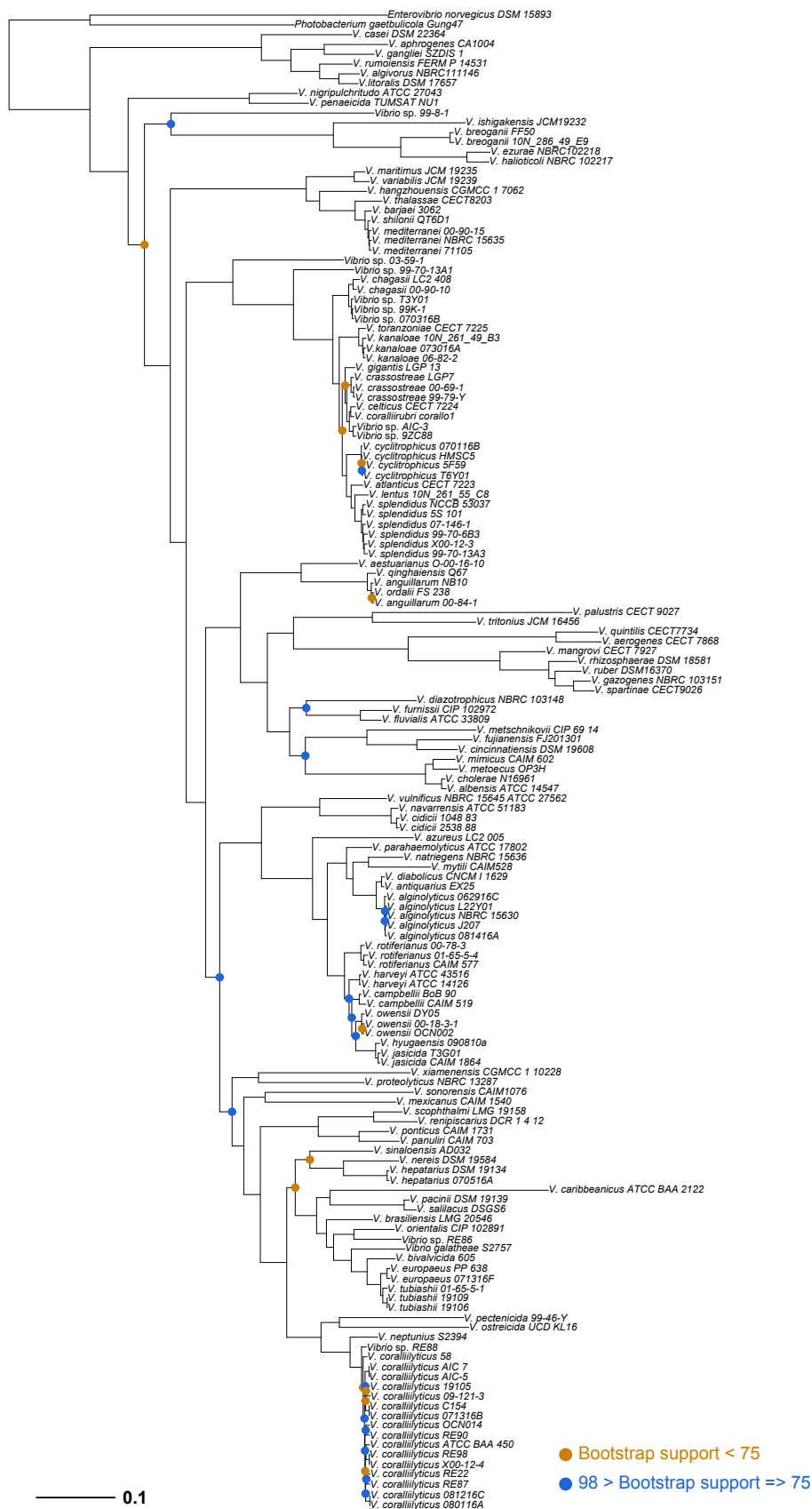

Supplement: Supplementary file 3 — Additional file 3 Fig. S2. Phylogenetic Tree of strains used in this study and reference strains. A concatenated alignment of 686 conserved amino acid sequences from single copy orthologous genes was used to construct a maximum likelyhood phylogeny. [file 12864_2020_6980_MOESM3_ESM.pdf]

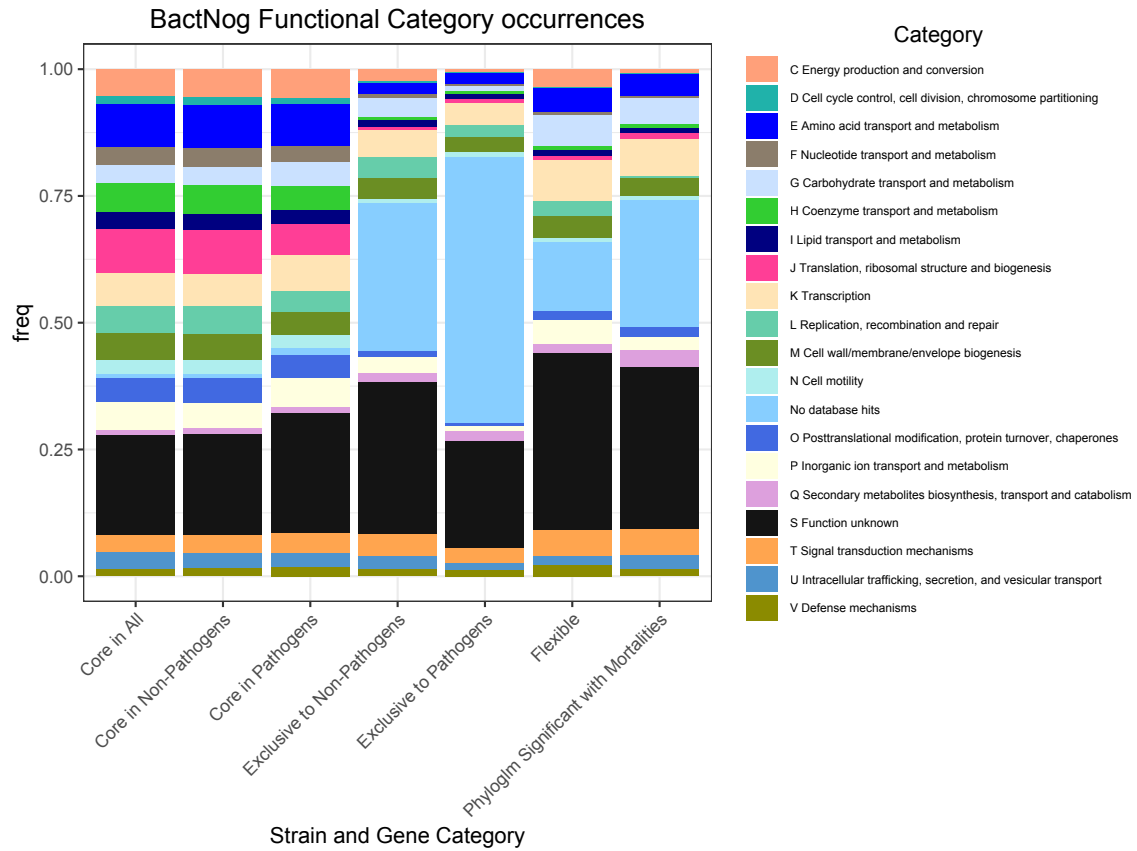

Supplement: Supplementary file 7 — Additional file 7 Fig. S4. Count distributions of bactNOG functional categories of genes among categories of different gene sets. The “Core in All” column refers to the core genome (n = 1693 gene clusters) of all strains (n = 51). The “Core in Non-Pathogens” column refers to the core genome (n = 1775 gene clusters) of all strains (n = 34) that are non-pathogenic (0.5 or lower mortality ratio). The “Core in Pathogens” column refers to the core genome (n = 2646 gene clusters) of pathogenic strains. The “Exclusive to Non-Pathogens” column refers to genes (n = 4010 gene clusters) that are found only in non-pathogens, but not necessarily conserved. The “Exclusive to Pathogens” column refers to genes (n = 1981 gene clusters) that are found only in pathogens, but not necessarily conserved. The “Flexible” column refers to genes (n = 3345 gene clusters) that are found at least once in both pathogens and non-pathogens, but are not conserved in either. The “Phyloglm Significant with Mortalities” column refers to all genes (n = 509 gene clusters) that had a significant correlation with pathogenicity towards C. gigas larvae. [file 12864_2020_6980_MOESM7_ESM.pdf]
