# Supplementary material for: Comparative genomic analysis of Vibrios yields insights into genes associated with virulence towards C. gigas larvae
Source: BMC Genomics. 2020 Aug 31;21:599. doi: 10.1186/s12864-020-06980-6 (PMC7457808; doi:10.1186/s12864-020-06980-6)
Supplement: Supplementary file 4 — Additional file 4 Fig. S3. Core and Pan-genome of 51 isolates in this study. (A) the core-genome represents all genes shared by each genome added. (B) The pan-genome represents the accumulation of all genes among all genomes with the addition of each genome. [file 12864_2020_6980_MOESM4_ESM.pdf]

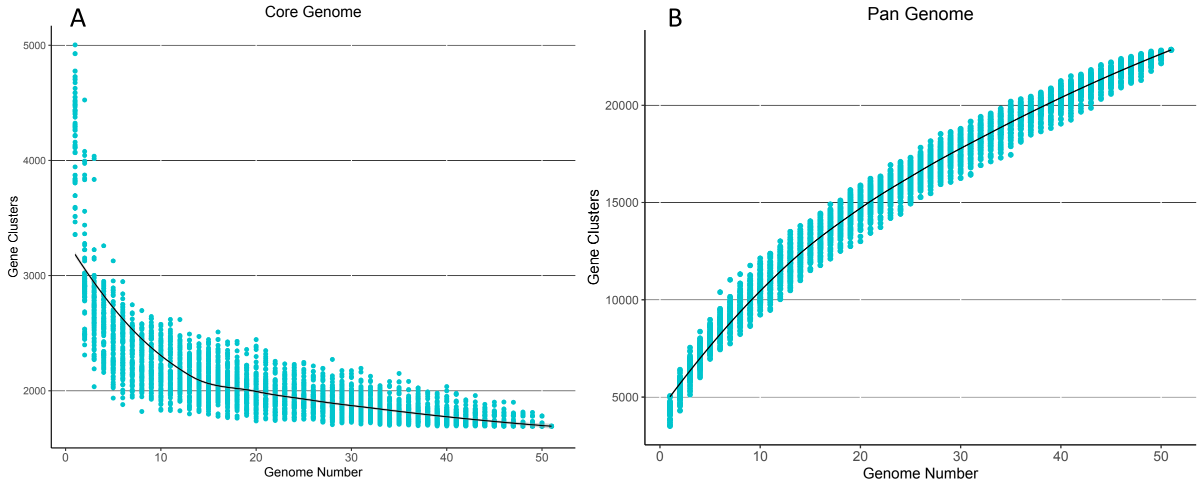

Supplementary Figure 3 Core and Pan-genome of 51 *Vibrio* isolates in this study. (A) The core-genome represents all genes shared by each genome added. (B) The pan-genome represents the accumulation of all genes among all genomes with each genome added.
