# Supplementary material for: Comparative genomic analysis of Vibrios yields insights into genes associated with virulence towards C. gigas larvae
Source: BMC Genomics. 2020 Aug 31;21:599. doi: 10.1186/s12864-020-06980-6 (PMC7457808; doi:10.1186/s12864-020-06980-6)
Supplement: Supplementary file 9 — Additional file 9 Fig. S6. Predicted phylogeny of Type 3 Secretion Systems using SctV amino acid sequences. Clades are color coded. Included in the phylogeny are proteins from tested isolates, specific Vibrio reference genomes, and from COG4789 comprised of more diverse reference genomes. Scale bar represents one change per amino acid. Tree was visualized with iTOL. [file 12864_2020_6980_MOESM9_ESM.pdf]

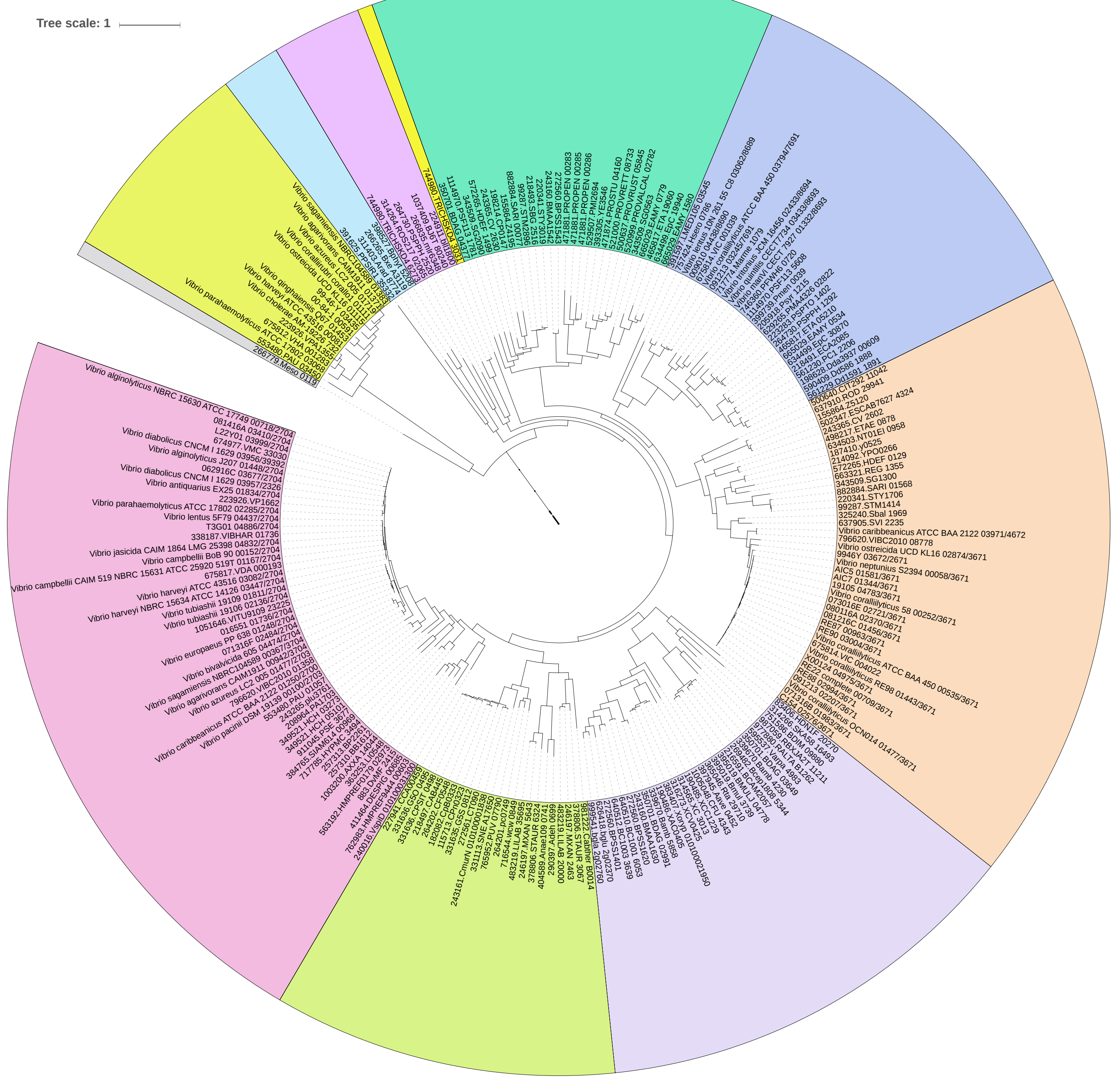

Supplementary Figure 7 Predicted phylogeny of Type Three Secretion Systems using SctV protein sequences. Clades are color coded. Included in the phylogeny, are tested isolates, *Vibrio* genomes, and proteins from COG4789 originating from a variety of reference genomes. Scale bar represents one change per amino acid.
