# Supplementary material for: Comparative genomic analysis of Vibrios yields insights into genes associated with virulence towards C. gigas larvae
Source: BMC Genomics. 2020 Aug 31;21:599. doi: 10.1186/s12864-020-06980-6 (PMC7457808; doi:10.1186/s12864-020-06980-6)
Supplement: Supplementary file 8 — Additional file 8 Fig. S5. Gene organization of the CPI-1 region within the complete genome of V. coralliilyticus RE22 and the draft genome of V. pectenicida 99–46-Y. Pink arrows indicate that the gene had a negative correlation with larval survival in virulence assays. Blue arrows indicate that there was no significant correlation with survival or that the gene was not tested. Yellow slashed lines indicate contig breaks on the CPI-1 of the V. pectenicida strain 99–46-Y draft genome. [file 12864_2020_6980_MOESM8_ESM.pdf]

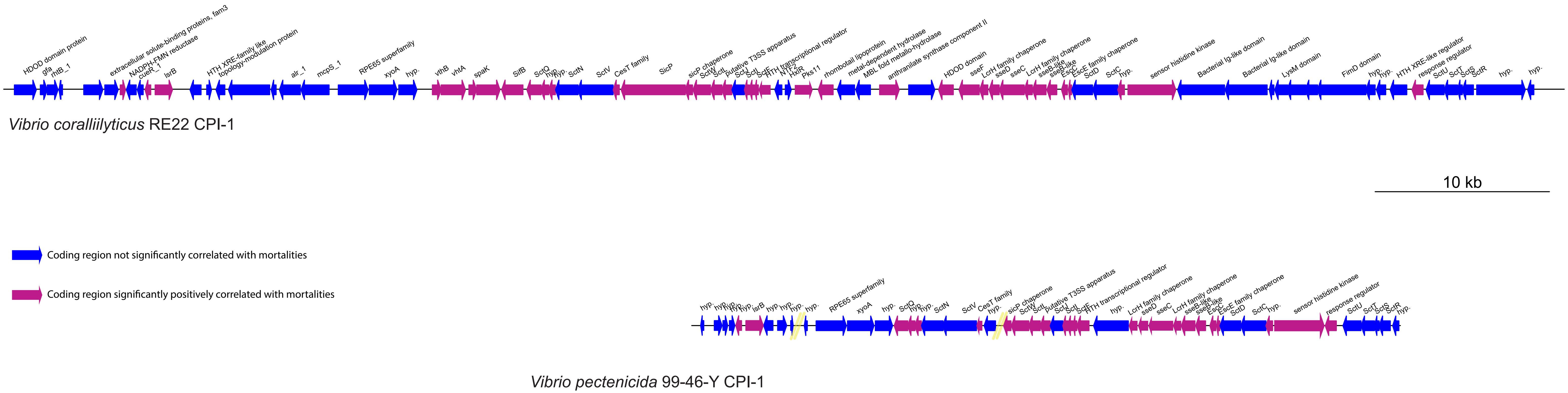

HDOD domain protein
gfa
rhtB_1
extracellular solute-binding proteins, fam3
NADPH-FMN reductase
cueR_1
lsrB
HTH XRE-family like
topology-modulation protein
alr_1
mcpS_1
RPE65 superfamily
xyoA
hyp.
vthB
vhtA
spaK
SifB
SctQ
SctN
SctV
CesT family
SicP
sicP chaperone
SctW
SctL
putative T3SS apparatus
SctJ
SctF
HTH transcriptional regulator
NTF2
HxIR
Pks11
rhomboid lipoprotein
metal-dependent hydrolase
MBL fold metallo-hydrolase
anthranilate synthase component II
HDOD domain
sseF
LcrH family chaperone
sseD
sseC
sseB-like
EscE
EscE family chaperone
SctD
SctC
sensor histidine kinase
Bacterial Ig-like domain
LysM domain
FimD domain
HTH XRE-like regulator
response regulator
SctU
SctT
SctS
SctR
Vibrio coralliilyticus RE22 CPI-1
10 kb
Coding region not significantly correlated with mortalities
Coding region significantly positively correlated with mortalities
Vibrio pectenicida 99-46-Y CPI-1
